# Early Metastasis of Clear Cell Renal Cell Carcinoma to the Esophagus: A Case Report

**DOI:** 10.7759/cureus.33635

**Published:** 2023-01-11

**Authors:** Heliberto Paez Quintero, Daniel Clavijo Cabezas, Nathalie Hernandez Hidalgo, Gregorio Londoño, Rafael Parra-Medina

**Affiliations:** 1 Oncology, Instituto Nacional de Cancerología, Bogota, COL; 2 Pathology, Fundacion Universitaria de Ciencias de la Salud, Bogota, COL; 3 Nuclear Medicine, Instituto Nacional de Cancerologia, Bogota, COL; 4 Pathology, Universidad Nacional, Bogota, COL; 5 Pathology, Fundación Universitaria de Ciencias de la Salud, Hospital San José, Bogotá, COL; 6 Pathology, Instituto Nacional de Cancerologia, Bogota, COL

**Keywords:** case report, anatomical places, clear cell kidney cancer, esophagus, metastatic

## Abstract

Metastatic involvement of clear cell renal cancer in the esophagus has been described in the literature as an uncommon condition. These usually present as late relapse causing clinical manifestations such as dysphagia and melena. We present the case of a 57-year-old man with a history of renal cell carcinoma who presented an early metastasis to the esophagus. In addition, we made a comparison of the reason for examination, time of relapse, and metastasis in other anatomical places of all the cases reported in the literature of esophageal involvement due to clear cell metastasis.

## Introduction

Metastatic involvement in the esophagus is a very rare finding, in most cases, it is caused by the invasion of adjacent neoplasms of other tissues such as the lung, trachea, and thyroid some metastasis in the esophagus through the hematogenous route occurs mainly from the liver and uterus tumors [1]. Neoplasms in the esophagus cause clinical symptoms such as dysphagia and melena, and for the study of the symptomatology of dysphagia the endoscopy of the upper digestive tract is one of the most important tools for diagnosis since metastases manifest as polypoid lesions or ulcerative lesions that extend beyond the submucosa [2].

The metastases secondary to clear cell renal cell carcinomas (CCRC) are present in approximately 30% of patients with advanced stages and this is associated with <15% 5-year survival [3]. The most common sites of metastasis are the lungs (70%) and bone (32%), on the other hand, the less frequent metastases are thyroid (0.7%) and gastric (0-7%), which represent <5% of the metastases of CCR [4-5].

Below we present the rare case of a patient with a history of CRC with metastasis in the esophagus.

## Case presentation

A 57-year-old man with a history of stage IV CCRC in the left kidney. The patient attended the consultation four months after the nephrectomy due to progressive dysphagia accompanied by a 15 kg weight loss. The endoscopic study revealed a 30-cm nodule with a firm consistency that involved 100% of the circumference and partial stenosis of the lumen of the distal portion of the esophagus, there is no good-resolution image of the endoscopy and it was not possible to repeat it due to the patient’s condition.

**Figure 1:**
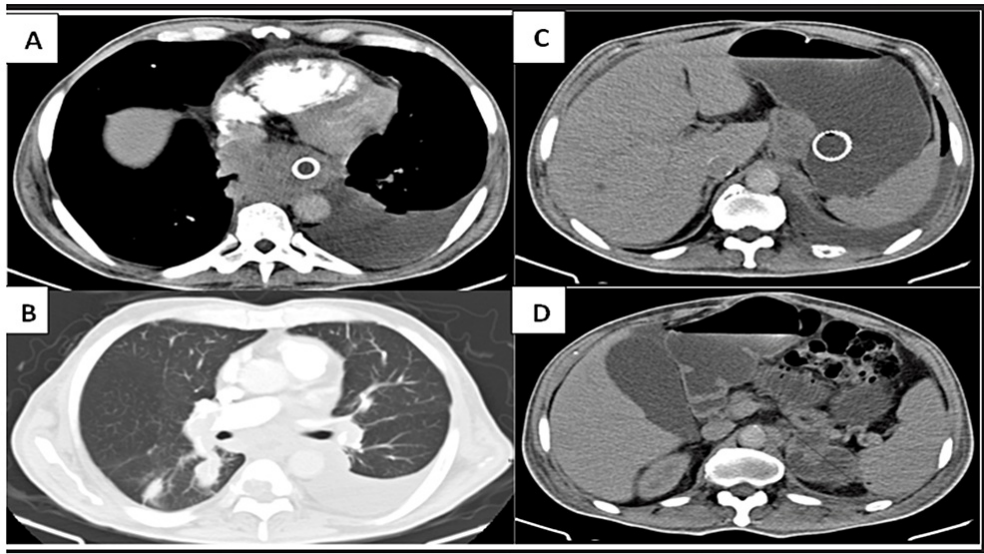
Chest and abdomen tomography. A) Mediastinal subcarinal mass involving the thoracic esophagus from the carina to the gastroesophageal junction with stenosis of the latter under stent management. B) In the lung parenchyma, solid nodular lesions are observed, some with mass criteria, especially in the upper segment of the right lower lobe related to metastatic involvement. C) In the liver, an indeterminate hypodense lesion is observed at the limit between hepatic segments V and VI of approximately 6.5 mm. Adenomegaly in the gastro-hepatic space of 45 x 37 mm. D) Mass behind the tail of the pancreas of the heterogeneous density of approximately 60 mm that seems to correspond to secondary involvement of the left adrenal gland and overdistension of the gallbladder.

The abdominal and thoracic tomography (Figure 1) revealed the presence of a mediastinal mass extending from the carina to the gastroesophageal junction with the compromise of the esophagus, liver and lung lesions, and left adrenal gland.

**Figure 2:**
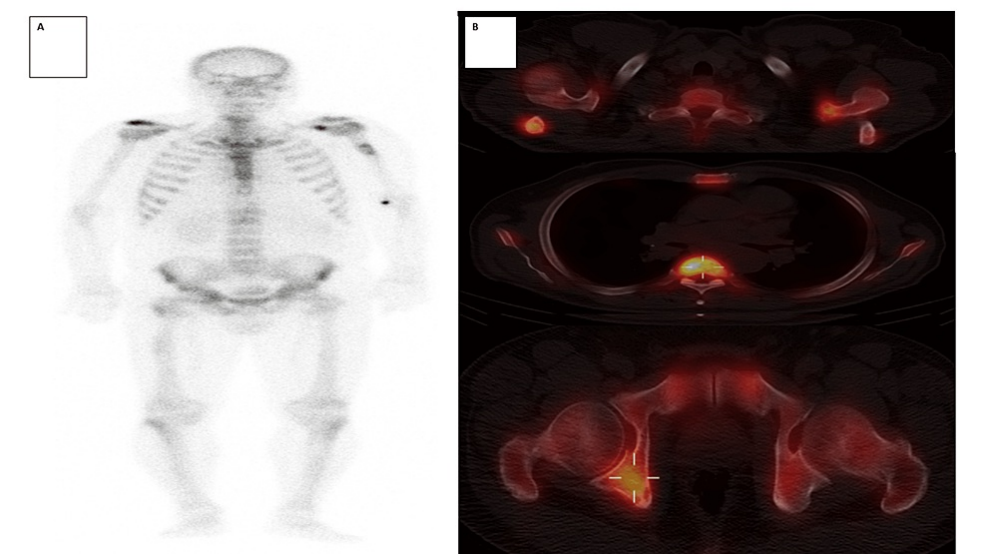
Bone scan with 99mTc-MDP. A) Whole body scan in anterior and posterior projection and SPECT/CT. High-uptake bone lesions are observed in the scapulae, proximal third of the left humerus, B) vertebral body of T5, eleventh posterior left costal arch, right ischium, and greater trochanter of the right femur secondary to metastatic involvement. MDP: methylene diphosphonate

Bone scintigraphy (Figure 2) revealed high-uptake bone lesions in the scapulae, left humerus, T5 vertebral body, eleventh left costal arch, right ischium, and right femur.

**Figure 3:**
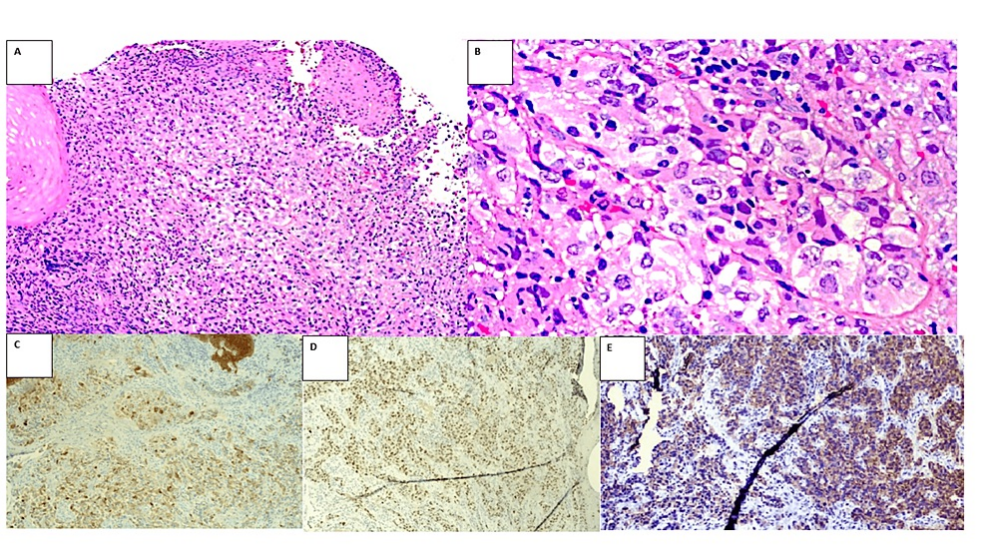
Histopathological study of the esophageal tumor. A) H&E 4x submucosa of the esophagus infiltrated by tumor cells, cells with clear cytoplasm, atypical nuclei, some with prominent nucleoli, B) mitotic figures are also observed. positive immunohistochemical studies: C) AE1/AE3 10X 3, D. PAX8 10X, E) CAIX 10X.

In the histological analysis of the biopsy taken at endoscopy, compromise of the esophageal submucosa was observed by malignant cells with clear cytoplasm, atypical nuclei, some with prominent nucleoli, which were positive in immunohistochemical studies for AE1/AE3, PAX8, and CAIX (Figure 3) and negative for CD10 and renal cell carcinoma (RCC).

The patient with an Eastern Cooperative Oncology Group (ECOG) of 1 receives 20 Gy external radiation therapy with palliative intent. Subsequently, an esophageal stent was placed. However, the patient presented dyspnea, pleuritic chest pain, spinal cord compression syndrome, and upper gastrointestinal bleeding, with submassive pulmonary thromboembolism, without improvement, and died days later. Unfortunately, due to the symptomatic and dramatic nature of the situation, the patient did not receive systemic therapy; however, a treatment option would be dual immunotherapy, taking into account that bleeding would not make it an initial candidate for management with a tyrosine kinase inhibitor. Regarding symptomatic management, radiotherapy could be considered as an option, however, in active bleeding, we consider direct endoscopic assessment to be a better option and, if possible, direct sclerotherapy or interventional radiology-guided management in the case of bleeding.

## Discussion

Esophageal metastases from CCRC of clear cell variants are even rarer. Seven cases have been described in the literature, including our case report, which are shown in Table 1 [1,2,6-10]. Esophageal metastasis of RCC is extremely rare and there is not clear evidence why they appear. One of the possibilities, continuity lesions are a plausible option, but directly in the available literature, it could not be verified as a clear trend of aggregation, so there remains uncertainty as to whether there is a pathophysiological mechanism that can explain it and for this reason, you should make the call to report more cases and be able to have a better idea of the associated causality.

**Table 1:** . Summary of cases reporting CCRC metastasis to the esophagus. CCRC: clear cell renal cell carcinoma

Author/Year	Gender	Age (Year)	Symptoms at diagnosis	Endoscopic finding	Time to relapse	Other metastasis sites	Systemic treatment/local therapy	
Cabezas et al., 2015 [1]	Male	38	Melena, anemia	Three polys of 7 mm	4 years	Lymph nodes, adrenal, stomach, lung	Systemic therapy didn’t described/local therapy with embolization	
								Izumo et al., 2015 [7]	Male	65	Dysphagia	Unique submucous polyps	10 years	Lymph nodes, lung	Observation with systemic therapy/surgical local		
																Ali et al. 2018 [8]	Male	82	Melena, dysphagia, and weightless	Mass of 5x4x7 cm^3^	13 years	Lung, lymph nodes	Sunitinib in systemic therapy/radiotherapy local therapy		
Padda and Si, 2019 [9]	Male	56	Dysphagia	Polyps 6 mm “Benign”	At diagnosis	Contralateral kidney	Observation with systemic therapy/surgical local																		
								Ohnita et al., 2021 [2]	Male	59	Asymptomatic patient	Unique submucous polyps	14 years	No available	Systemic therapy not required/endoscopic surgery		
																Yasuoka et al., 2021 [10]	Male	68	Asymptomatic patient	Tumor *único en el esófago parte* inferior	9 years	Lung, Lymph nodes	Systemic therapy with axitinib/surgical treatment		
This case, 2022	Male	57	Dysphagia and weight loss	Mass 30 mm obliteration complete	4 months	Lung, Lymph nodes, Adrenal	Systemic therapy was not received/radiotherapy local																

The most common reason for consultation in all patients was dysphagia presented in four patients, weight loss and melena were also frequent in two patients. Particular attention was that all reported cases are women, in addition, a large percentage were over 50 years of age. Five patients had a history of CRC, however, there was one case where the initial diagnosis of CRC was metastasis in the esophagus and six cases have metastases to other parts of the body [9].

The time to metastatic relapse is variable, usually several years after surgical treatment. Our case report has an early filing of 4 months, which is below the average (24 months) in a series of cases [1] and for other case reports [2,7-9], also the initial size of the mass is unusual with a commitment of 100% of the lumen, which is greater than the reported cases, where a solitary nodule or polyp is generally reported that does not occupy the entire esophageal diameter [2,9,10], the treatment is multidisciplinary, with surgical treatment as the first option and a combination of radiotherapy and chemotherapy [8,9], with the increasingly widespread use of tyrosine kinase inhibitors such as pazopanib [2-6], or checkpoint inhibitors.

## Conclusions

Metastatic involvement in the esophagus by CCRC is extremely rare and only six cases have been described. The present case is interesting due to the time of appearance of the metastasis and the endoscopic size observed and compared with the other reported cases and allows us to remember that the most usual case of a neoplastic commitment in a patient with an aggressive cancer is possibly a metastatic disease that a second primary of different etiology and histology.
